# Together but still alone - A qualitative study exploring how family members of persons with incurable oesophageal or gastric cancer manage everyday life

**DOI:** 10.1186/s12904-024-01576-3

**Published:** 2024-10-26

**Authors:** Sofia Kårmark, Marlene Malmström, Jimmie Kristensson

**Affiliations:** Lund university, Box 117, Lund, 221 00 Sweden

**Keywords:** Family members, Oesophageal cancer, Gastric cancer, Early palliative care

## Abstract

**Background:**

Cancer affects not only the person with the disease but those around them. Being a family member is described as strenuous and, often, associated with stress, anxiety and feelings of loneliness. There is a heightened risk of distress for family of those with fast-progressing, severe oesophageal or gastric cancer. Early palliative care involving family is vital yet often overlooked. In order to include family members in early palliative care their management in everyday life needs to be explored.

**Method:**

Qualitative inductive interview study using content analysis guided by Graneheim and Lundman.

**Result:**

The analysis resulted in the overarching theme “Managing the disease together but still alone”. Three categories were identified: Adapting to the disease, Taking control of the situation, Processing emotions. Each category described family members management in various aspects of everyday life together with the ill person and alone.

**Conclusion:**

The results may contribute to an awareness of family members’ management of large parts of everyday life and, further, their feelings of loneliness, and indicates that family members should be included early in oesophageal or gastric cancer palliative care. Further studies are needed to develop the content of such family-inclusive early palliative care.

**Supplementary Information:**

The online version contains supplementary material available at 10.1186/s12904-024-01576-3.

## Summary box

**What is already known on this topic**

Cancer is considered a “we-disease”. Being a family member or informal caregiver of someone with cancer is fulfilling but strenuous and family members of those with oesophageal or gastric cancer have an increased risk of prolonged stress. Approximately half of all cancers of the oesophagus or stomach are incurable and the disease course is rapid, with severe symptoms. It is important to include family members in palliative care, which ought to be initiated early. There is currently insufficient knowledge about how family members of persons with incurable oesophageal or gastric cancer manage in everyday life, and also about the palliative care including them that should be provided.

**What this study adds**

This study adds an insight into family members’ perspective of incurable oesophageal or gastric cancer and how they manage everyday life. It provides information about their management of everyday life, early in the disease course and on many levels, both together with the ill person and alone.

**How this study might affect research, practice or policy**

The study could contribute to an awareness in the health care community of how family members manage the disease, which might lead to palliative care inclusion. It also might inspire further studies on how the palliative care could be tailored to family members of those with incurable oesophageal or gastric cancer.

**Supplementary Information:**

The online version contains supplementary material available at 10.1186/s12904-024-01576-3.

## Introduction

Cancer affects family members in several ways and is described as a “we-disease” due to its capacity to engage collectively and lead to family members’ involvement [1]. During the course of the illness, family members become informal caregivers who supports, assists and attends the ill person´s needs [2]. This role has been described as both rewarding and fulfilling, but it is also demanding and strenuous leading to stress, anxiety and feelings of loneliness [1, 3–5]. Family members take on several roles that change as the ill person transitions between disease stages, such as provider, companion and decisionmaker. Studies report that family members experience a lack of support in balancing these roles and that spouses have an increased risk of developing psychiatric disorders because of the strenuous everyday life as a caregiver [6, 7]. There is a documented increased risk for family members of those with oesophageal or gastric cancer to experience prolonged emotional distress [8].

About 1,300 patients, typically men above 65 years, are diagnosed with oesophageal or gastric cancer in Sweden yearly [9–11]. The majority (60%) already suffer from an incurable disease at diagnosis due to comorbidity and/or advanced disease, and an estimated 80% have died within 1 year of diagnosis. The disease course is characterized by several severe symptoms associated with nutrition and quality of life [11–14] and it is reported that those who are most ill utilize more health care [15]. Realizing that everyday life might significantly change put a strain on family members [16, 17]. Everyday life can be defined as the mundane life and routines that one often take for granted such as talking, working, eating, cleaning, shopping, resting and so on [18]. The overall situation presents new lived experience which makes it difficult to be prepared and forces family members to find ways of managing. “Family management in cancer care” refers to family caregivers’ way of both consciously and unconsciously managing the illness in everyday life, which may include stress management, supporting the patient in managing the illness, managing symptoms and self-care activities [19, 20]. Coping strategies on the other hand are subconscious acts rooted in defence mechanisms to tolerate or reduce stress [21, 22].

In this study a family member is the person selected by the ill person and can be the spouse, the long-lived partner, or children and/or other relative. More knowledge is needed about family members’ lived experiences specifically relating to incurable oesophageal and gastric cancer. To begin to comprehend their situation it is necessary to qualitatively explore their lived experiences of management. The exploration of how they manage in everyday life could then indicate further studies of how they cope and which coping-strategies that are used.

A person who is close to someone with incurable oesophageal or gastric cancer should be introduced to palliative care. Palliative care according to the World Health Organization (WHO) is a person- and family-centred approach to meet the needs of those with incurable disease or suffering and their families and aims to support them throughout disease course. Palliative care includes physical, psychological, social and existential components of everyday life [23]. The International Association of Hospice and Palliative Care (IAHPC) also emphasizes the importance of communicating with family members [24]. It is evident that palliative care should be started early and be integrated. Early palliative care is beneficial for those with incurable disease as it gives better symptom management and increased quality of life, for both those with incurable oesophageal cancer and gastric cancer [7, 25–27]. However, for it to be successful the early integrated palliative care has to be tailored to the illness group and diagnosis, not solely relying on research concerning advanced cancer in general but targeting specific symptoms and needs, and palliative care components related to each patient group and their family members [28]. In this regard family members’ perspectives have been insufficiently researched [7, 25, 27], especially among this patient group. It is important to explore how family members manage the situation to learn how to include them in early palliative care.

Being a family member of a person with cancer is described as engaging, strenuous and challenging and family members of persons with oesophageal or gastric cancer have an increased risk of prolonged distress [1–5]. Early palliative care for incurable disease of the oesophagus or stomach is of essence due to the rapid disease course, great symptom load, decline in quality of life and the increasing health care utilization for the most ill. The early integrated palliative care should be family-inclusive but necessary specific diagnosis content concerning family members’ experiences is insufficient [7, 23–25, 27, 28]. A greater understanding of family member’s lived experiences and an exploration of their everyday management is needed to begin to develop family inclusive early integrated palliative care for oesophageal and gastric cancer. The need for more knowledge motivates the question and the result could contribute to increasing the knowledge as well as the development of early integrated palliative.

## Aim

To explore how family members to persons with incurable oesophageal or gastric cancer manage everyday life.

## Design

This is a qualitative inductive interview study [29].

### Setting

Sweden has a public tax-funded health care system consisting of 21 health care regions across the nation [30]. Each region treats cancer patients. Oesophagus and gastric cancer is defined as specialized care through a national treatment assignment; family members’ needs are addressed in the assignment in terms of providing good information.

Patients visits the assigned centres for discussions with surgeons and nurses about the diagnosis, interventions and possible palliative treatment plans. Regional oncological clinics perform further possible care by referral. Family members are often encouraged to partake in visits; however, routines and plans for assessing and meeting family members needs are often lacking. During 2020–2022 Corona pandemic restrictions prevented family members from physically accompanying their relative on health care visits.

### Participants

Patients diagnosed with incurable oesophageal or gastric cancer and already participating in a study focusing on patients’ needs, or identified by nurses, were approached at the specialized clinic and asked if they had family members who might be interested in participating in the study. Family members were thereafter contacted, informed about the study and invited to participate. Inclusion criteria were being a family member of a patient with incurable oesophageal or gastric cancer, being ≥ 18 years of age, having no significant cognitive impairment, and being fluent in Swedish. With the assistance of nurses at the assigned clinic, participants were purposively and strategically selected during 2018–2022 to ensure as much variability in disease trajectory and relationship with the patient as possible (Supplementary file). The sample was intentionally evenly spread from a month after the next of kins diagnosis to after their death in order to capture as wide range of experiences as possible. Different types of family members were approached for participation, however most family members participating were female, reflecting the typical patient being male in a heterosexual marriage. Altogether 21 family members were approached, five of whom declined resulting in 16 family members (-15 females and one male) participation, twelve of whom were wives of patients. A variation between diagnosis was also tried to be achieved, consequently eleven persons had oesophageal cancer and four had gastric cancer. Written consent from both patients and family members was collected and family members were contacted by phone for interview scheduling.

### Data collection

In trying to find lived experiences of management in everyday life, data was collected by in depth interviews. Family members were interviewed by the first author (RN, PhD candidate, female) with previous experience in interviewing patients with severe disease. The interviews (*n* = 15) were conducted at different stages of the disease trajectory ranging from 1 month after diagnosis to after the patient’s death, to cover management at different stages of the disease course. The participants chose whether the ill person was present during the interview. Ten interviews were performed with both the patient and their relative present, five with only the family member, and one with two family members. One participant was interviewed twice, once together with the ill person and once on their own. The interviews were audio-recorded and ranged from 50 to 100 min. All interviews (except one which was carried out over the phone) were done face to face and were conducted either in the family members’ home or at the hospital.

The interviews followed a semi-structured interview guide with open-ended questions developed for this study, not published elsewhere (Supplementary file). The first interview was conducted with an experienced colleague (KD; RN, PhD candidate, female) to ensure appropriate technique and meeting objectives. After 15 interviews the data were listened to and read through. The data collection was deemed sufficient when nothing further seemed to emerge from the text.

### Data analysis

Content analysis is a method suitable for finding emerging themes from a text with systematically coded data. The aim of exploring lived experiences directed us towards qualitative analysis which is appropriate in trying to find latent patterns and generate themes. Data were consequently analysed using conventional content analysis as described by Hsieh and Shannon [31] and guided by the analytical steps described by Graneheim et al. [32] and Graneheim and Lundman [33].

The first author (SK) transcribed the interviews to ensure both the method’s rigour and content knowledge. All interviews were listened to and read through multiple times for to gain sense of the whole. Systematically meaning units were identified keeping the aim in mind, condensed into abstracted units, codes derived from the condensation of the text and were not predetermined. Latent patterns were searched for within the codes by reading and reflecting on them several times and grouped into categories and subcategories according to the patterns. Based on the categories an overall theme generated describing the abstracted collection of the interpreted text (Table 1.). Each step of the process was discussed and collaborated on with the last (JK; RN, associate professor, male) and second author (MM; RN, associate professor, female) leading to consensus. Participants quotations followed by individual code numbers are presented to illustrate the findings. Reported according to COREQ checklist (Supplementary file).

**Table 1:** Description of the analytic process

Meaning unit	Code/condensed meaning unit	Subcategory	Category
“We have tried many soupes, but it’s nothing we like.”	Experimenting with food	Trying to maintain normality	Adapting according to the disease
“We have written legal documents now but if we hadn’t done it, I would’ve panicked. And now I don’t know how long he lives and then we have to do it.”	Writing legal documents to avoid panicking	Planning for the future	Taking control over the situation
“X has an idea, what if I can get the operation. I’m thinking, you won’t get it. But he may gladly hope so, and so do I too somewhere.”	Allowing the ill person to hope for an operation	Trying to maintain hope	Processing emotions

Ethical considerations.

## Result

**Fig. 1:**
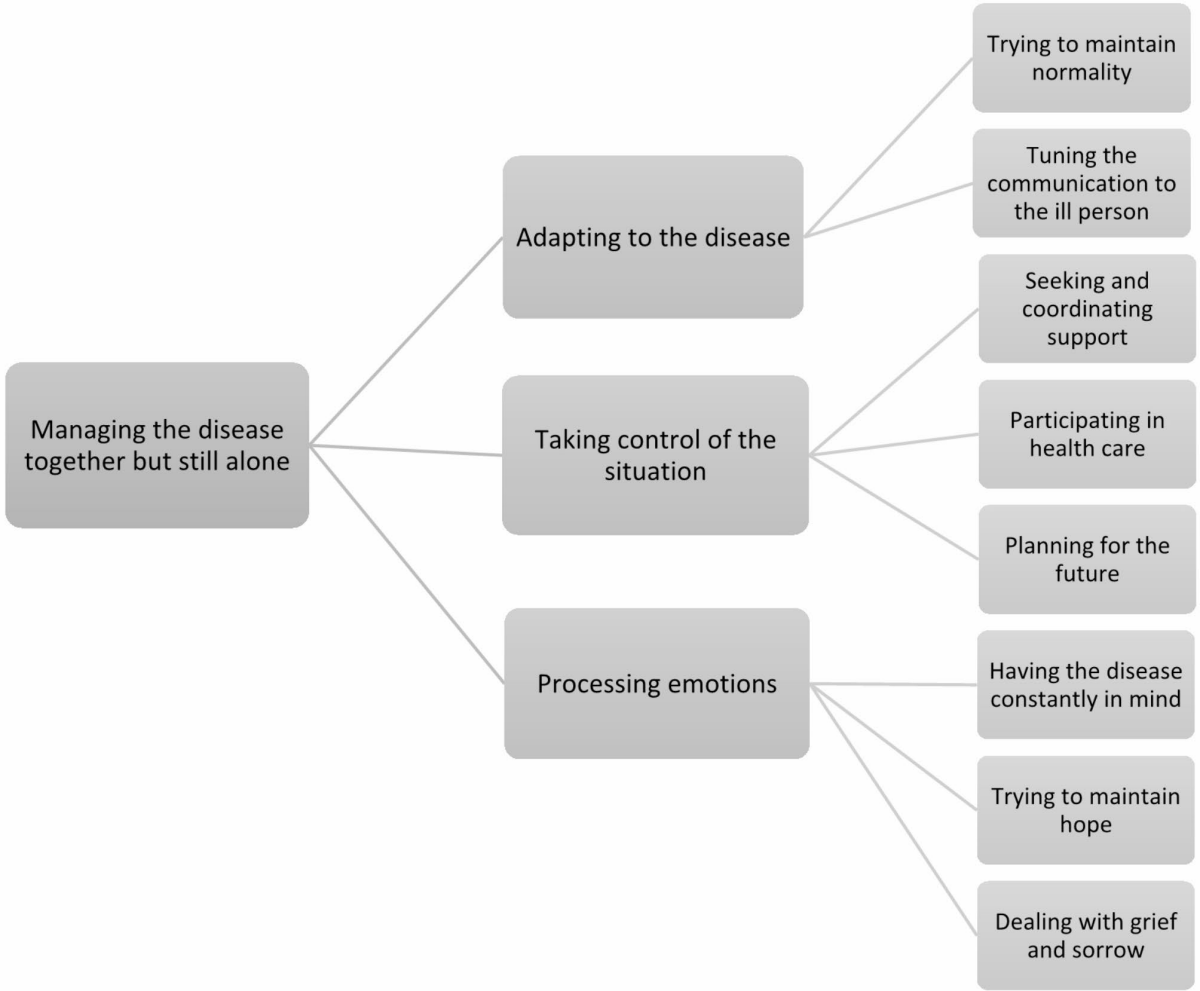
The identified theme, categories and subcategories

The analysis revealed that the way family members to persons with incurable oesophageal or gastric cancer manage everyday life was captured in the overarching theme *Managing the disease together but still alone*. It was highly visible that the disease had changed life to a great extent and that it required them to adapt management in practical, social, and emotional parts of everyday life. They found themselves in the situation without no choice, and taking control was therefore essential. However, they actively chose to be involved not leaving the ill to fender for themselves. In ways family members managed everyday life together with the ill, but often they had to manage the situation on their own. Simultaneously the family member could on the one hand manage together with the ill person, but at the same time were alone in managing things they did not want to burden the ill person with and with their own experiences and emotions. The overall theme comprised three categories with associated subcategories that describes the content of the theme from various perspectives (Fig. 1).

### Adapting to the disease

One significant feature of managing everyday life was having to adapt to the disease. Adapting meant altering certain aspects to keep what was deemed necessary or what constituted a normal everyday life. Making both physical and mental room for the disease and the ill. Also, doing things differently and deciding what or when something needed to be done together with the ill or was solvable on their own. The category comprised the subcategories: *Trying to maintain normality and Tuning the communication to the ill person.*

#### Trying to maintain normality

Trying to maintain normality was described in various ways. A common feature was adapting details or parts of everyday life to keep it as normal as possible, which was visible throughout the disease course while considering circumstances caused by the disease. Family members reasoned with the ill about topical adjustments and how to make them. Jointly they adapted meals, household work, routines and social activities. They tested and evaluated alternatives to find suitable adjustments.

*On Fridays and Saturdays he always has a glass of beer and I have a glass of wine. We can’t do that anymore of course*,* but I’m stilling having mine. I thought*,* water is just boring. So I bought Christmas soda* [*julmust*, a carbonated, non-alcoholic beverage made from malt], *and that does the job. One glass. It makes for something a bit different.*

(No. 13)

Family members expressed the importance of putting the ill person’s needs first. Together they rearranged their plans according to treatments or other disease-related activities. Great effort was made to keep meaningful joint activities only altering them in such a way as to enable the ill’s participation, like arranging theatre visits according to the ill’s condition. They saw this as a significant factor in trying to maintain normality.

Family members reflected on, often alone, which aspects of their everyday life needed to be adapted. They would think of or experiment with new adjustments before presenting them to the ill. For example, one family member thought and discussed with her boss how to improve her availability to the ill person while working, which led to changing assignments and her working from home most days before eventually presenting the adjustments to the ill. On the other hand, family members tried to maintain normality by compromising their individual activities such as swimming, thus not forsaking their own quality of life.

#### Tuning the communication to the ill person

How to communicate about the ill and the situation was managed by tuning the communication to the ill person’s current condition. A mutual approach that suited the ill was discussed between the family member and the ill person, often leading to an openness about the situation and the ill person’s health towards family and friends. Talking freely was one way of dealing with having an incurable disease and suited several of the ill persons. However, some preferred not to talk about the disease at all; to them communicating about it made the disease more present, causing sadness and despair. In those cases the family members followed suit.

[Interviewer: It was just you guys in the family who knew? ] *Yes*,* of course. And we have to respect that. Yes*,* also my job*,* I was compelled to tell. But they are unaware*,* they don’t know who he is.* [Interviewer: But it’s what he wanted, he wanted to be left in peace? ] *He said*,* “Let me be.” Yes*,* we respected … We had to do that.*

(No. 11)

Family members would also on their own tune the communication to the ill person. When it was jointly decided not to communicate about the situation, some family members would solitarily find other ways to not compromise the ill’s wishes, which included talking to neighbours or close friends alone or to personnel during health care visits. Also to avoid causing distress or sadness to the ill person, some family members would decide not to talk about the illness with them. All participants would tune the communication to the ill person albeit in different ways.

### Taking control of the situation

One way of managing was taking control. Family members actively participated and shared the situation with the ill person. Partaking actively in everyday life with the ill person but managing many aspects on their own created a sense of control. Most did not trust the ill to reliably provide them with information. To be on top of things they instead took control by making sure they were well informed. They also planned and arranged to meet the needs of the ill, themselves and others.

The following subcategories describes how managing by taking control created a sense of security and served as a comforter: *Seeking and coordinating support*,* Participating in health care and Planning for the future*.

#### Seeking and coordinating support

Family members managed support together with the ill person and on their own in different ways. One way was coordinating in-depth conversations within the family both with and without the ill person. When in a married or long-term relationship, partners often verbally expressed the need for and received have emotional support within the relationship. The family member coordinated activities for joint support, like professional counselling meetings but also nature walks or gym sessions. When seeking out support for themselves it seemed important for the family members to interact with someone who could understand the situation. They might turn to close family, friends, neighbours, or acquaintances with similar experiences. One participant joined group sessions for family members of terminally ill for invaluable support. Others found consolation in a pet’s unconditional affection.

*And this person*,* whom as I say I go out and take walks with*,* she’s fantastic. She has to deal with a lot. Yes*,* we do talk a lot. And what we say we have decided will stay on the jogging path.*

(No. 14)

#### Participating in health care

One aspect of being in control was participating in the ill persons interactions with health care. This included together attending hospital visits, treatment appointments and conversations with health care personnel. Another way was keeping a “cancer journal” specifically for disease-related information such as treatments, medications or health care contacts. Family members also discussed, planned and took on disease related self-care activities with the ill person, like tube feeding and medication administration. They organized treatment transportation, making sure the ill person would make appointments and would not need to wait outside for public transportation. Family members were involved in all aspects of the ill’s health care, albeit not at all times yet creating a sense of control.

Family members often considered themselves carriers of specific knowledge of the ill persons’ health and needs and thought it important to be able to provide it when needed. Since not trusting the ill person with providing necessary information some worried about not knowing disease activities or progression and feared the ill person’s incapability of understanding and communicating necessities. They managed this lack of trust and fears by taking control and making sure to be a part of the health care process.

*Because I’m the one who has to give the medicine*,* with the help of X himself to start with of course. But later it turned out that I had to keep tabs on him and what he had taken and not taken. And we sit and have a meeting where we talk about which different pain medications*,* different medicines they will be initiating and discussing; then I want to be there at the meeting and give my opinion and say*,* “Yeah*,* no*,* that Oxynorm wasn’t good before he was going to eat*,* because …”*.

(No. 5)

#### Planning for the future

One important part of taking control was planning for the future. The family member planned for future scenarios ahead in the disease course or life without the ill person. Together, however, they would prepare documents, economy or housing adjustments. They made practical arrangements regarding chores like lawn mowing and car driving, but also community health care assistances and aids such as wheelchairs and beds. On the other hand, the prospect of taking on chores previously done by the ill person often led to anxiety. Some would ask the ill person to show them or talk them through a process so that they would be able to cope in the future. Moreover, certain planning had to be done without the ill person, like what to do with their belongings and how to honour them after their death.

*How are we to address the future? Well*,* we don’t know. It’s sort of like this*,* it’s a ridiculous example*,* but for example*,* where is the water faucet? That thing that turns off the water. […] I know it now*,* but where is it? So it’s these sorts of practical things that I want to know.*

(No. 15)

### Processing emotions

Family members described experiencing a large range of emotions that had to be managed, like fear, despair, sadness or helplessness but also hope and solace. They recounted difficulties to disconnect from many of these emotions in everyday life making management inevitable. Processing emotions was sometimes not even a choice but happened without control. This category comprises the subcategories: *Having the disease constantly in mind*,* Trying to maintain hope and Dealing with grief and sorrow*.

#### Having the disease constantly in mind

In everyday life with the ill person the disease was constantly present. The disease would often dictate or influence decision making, decisions such as which activities to do and how.

*There is a third party in our relationship*,* the cancer.*

(No. 6)

Expressed by a family member describing the new constantly present “companion”. Even when not mentioning it and doing ordinary things like going shopping or walking the dog, the disease lingered in the back of everyone’s mind. Accepting the existence of this new companion was a form of management. But having the disease constantly in mind awoke emotions that had to be processed, like the inability to enjoy everyday life or the disappointment over unmet expectations or goals. Another way of managing was getting used to the emotions by letting the disease in, consequently familiarizing themselves with the disease and thus improving emotional processing. On the other hand, some family members felt helpless having the disease constantly in mind as this forced them to process emerging emotions.

#### Trying to maintain hope

Trying to maintain hope proved to be essential for family members management. Together with the ill person they would maintain faith in the effect of treatments - to the extent that despite the diagnosis and their understanding that the disease was incurable, they still hoped for survival and that the next scan would show a positive picture. They would discuss with the ill person the importance of maintaining hope and positivity for each other’s wellbeing, and even for slowing or reversing the disease progression. They would mutually convey a hopeful appearance towards family and friends and provide a positive atmosphere. The family members privately would make a mental note of positive developments or consider a lack of symptoms as an indication that the disease was less rapid or severe than predicted. For example, a reduction in painkillers per day made one wife believe that her husband was not as ill as previously thought.

*I don’t want to think about … For that matter*,* it’s not at all certain that he will go before I do. It’s not certain at all. Anything can happen. People drive like idiots*,* and so on*,* so no*,* I won’t think that way*,* that I will end up alone. Someone will*,* but … no.*

(No. 10)

#### Dealing with grief and sorrow

When processing their emotions, family members dealt with grief and sorrow in individual manners. However, most had a need for an outlet of such whether it was together with the ill or alone. A wide range of ways of dealing with emotions was described; acknowledging them as a part of the process was an important aspect of dealing with them.

*We have said this from the beginning. That you have to be allowed to be sad*,* otherwise you can’t get happy again.*

(No. 8)

Some managed by sharing the emotions, for example by crying together. Others put their feelings on hold. They talked with the ill about how not to let them affect their mutual quality of life and to put them on hold until they became unavoidable. Family members had to manage these emotions on their own as well. They would cry alone, sparing the ill person, or read literature to understand and learn how to process their feelings. Some would manage by denying the feelings’ existence, and a few explained that there was no reason to deal with grief and sorrow as they were not sure the ill person would pass away from the disease.

## Discussion

This study presents an insight into how family members of those with incurable oesophageal or gastric cancer manage everyday life. In the exploration of they managed, the overarching theme of being in the situation together yet feeling alone became apparent. The results contribute to a deeper understanding of how family members manage everyday life, and provide implications for early palliative, underlining the need to provide support and interventions to family members.

### Result discussion

The results clearly revealed that family members had to manage large parts of everyday life. A significant portion were adjusting in accordance with the ill person, such as changing meal routines or social communication. Managing everyday life began directly after the diagnosis and substantially affected family members, often resulting in solitary strategizing. Studies show that family members are involved in strategizing for and making adjustments in all disease-related areas [1, 2, 17, 34], and that family members of those with advanced cancer manage their situation by making practical arrangements in everyday life [35]. In alignment with previous research, our results show that family members of those with oesophageal and gastric cancer too manage by strategizing and adapting large parts of their everyday life starting early on. This indicates the importance of involving them in early palliative care [7, 25–27].

One striking finding was that family members managed everyday life together with the ill person but experienced being alone. This was particularly prominent in the category “Dealing with grief and sorrow” where family members mentioned needs for acknowledgement, and confirmation regarding their everyday management. They found support from family and friends, but no from health care. Previous reports reveal loneliness among family cancer caregivers and lack of emotional support [36, 37]. Providing family members with tools to manage emotions may contribute to a reduced sense of being alone, as demonstrated in a therapeutic group-based intervention study where cancer-bereaved adults presented short-term improvements in grief and depression [38]. There is a review that implies how professionals such as nurses should help informal caregivers of incurably ill prepare for the grief and bereavement to come, and indicates the importance of policies providing resources to enable home-based grief support for informal caregivers in order to manage their grief during challenging times [39]. The findings of our study indicate that, when managing emotions such as grief and sorrow, family members of those with incurable oesophageal and gastric cancer need support to address loneliness and grief. Research show that support such as therapeutic group-based or home-based interventions might be appropriate.

Growing numbers of cancer cases mean that more people than ever before are forced to find ways of managing everyday situations in cancer [40]. Numerous studies reinforce the importance of considering family members as part of the disease course. For instance, reduction of anxiety and stress is associated with family members partaking in palliative care planning [41, 42]. However, in this study participants were already in a way part of this process since they were greatly involved in all health care aspects of the ill person. The positive effects of proactive health care are well known. The current findings could inspire proactivity through targeted information and care planning. Research presents psychoeducational models as successful concerning informational needs [43], but it is critical to further develop interventions addressing feelings of loneliness [37]. Loneliness is described as a distressing feeling connected with perceived social isolation [44, 45]. In this context, positive experiences for informal caregivers to cancer patients have been reported concerning digitally based support [46]. A digital tool may be a feasible proactive option to increase support and information accessibility without further straining health care resources. One study explored the intervention of a multidisciplinary early palliative care (EPC) team for patients and caregivers, providing holistic support, guidance in decision-making and preparation for the future. Both patients and caregivers felt supported and guided by the team in their illness experience. Although a small study the result provides an implication of how interventions could be organized to target the support needed for family members [47]. On the other hand, health care systems might not have the ability to be organized accordingly, palliative care should perhaps instead be provided throughout the health care system and not solely by specialised teams in order to capture family members early physical, psychosocial and existential needs. For this to be possible clinicians needs educational efforts and tools provided for interventions. However, further studies are needed for specific proactive interventions concerning family members’ everyday management in relation to oesophageal and gastric cancer.

### Methodological considerations

Together with the aim the research group’s a long experience with content analysis and how it provided a distinctness in systematically moving towards a latent result for the first author who was a PhD-student, motivated the chosen method. Concepts of qualitative trustworthiness includes credibility, dependability, transferability and confirmability [32, 33, 48]. Credibility was strengthened through participant variation, with a sufficient number of participants, reflecting the demography, leading to a wide perception of management. A strength was the ill person participating in most interviews. As management was largely performed together the process was more easily recounted together. However, singularly some family members revealed further emotional management and experiences of loneliness. A robust description of the analysis is presented, and the authors strove for consistency in the findings through triangulation and consensus. Awareness and consideration of the researcher’s preunderstanding and experience of upper gastrointestinal ensured dependability. However, no fieldnotes existed and no participant feedback was enabled.

Transferability has been achieved by providing descriptions of the context, participants and selection process together with the findings. The results reflect the data, which affirms confirmability. Describing the method and presenting the of abstraction, interpretation process and quotations in the text enhances authenticity. A clear presentation by category and theme and meeting the aim while not discarding diverse cases also underlines this. Rigour is additionally systematically reported through The COREQ checklist.

## Conclusion

This study implicates that support aimed towards everyday management and feelings of loneliness should be offered to family members of those with incurable cancer in the oesophagus or stomach and suggests that they should be included in early palliative care. Further studies of the content are needed.

## Electronic supplementary material

Below is the link to the electronic supplementary material.

Supplementary Material 1

## Data Availability

The data analyzed during the current study are available from the corresponding author on reasonable request.
