# Supplementary material for: Together but still alone - A qualitative study exploring how family members of persons with incurable oesophageal or gastric cancer manage everyday life
Source: BMC Palliat Care. 2024 Oct 26;23:249. doi: 10.1186/s12904-024-01576-3 (PMC11515144; doi:10.1186/s12904-024-01576-3)
Supplement: Supplementary file 1 — Supplementary Material 1 [file 12904_2024_1576_MOESM1_ESM.docx]

# “Together but still alone - a qualitative study exploring how family members of persons with incurable oesophageal or gastric cancer manage everyday life.”

## Interview guide.

|  |
| --- |
| Do you want to tell me a little bit about who you are? |
| Can you tell me something about what had happened before we spoke, what made you and/or your family member suspect that he/she wasn’t feeling well or was ill? |
| Tell me, how did you and/or your family member go about seeking help and then finally getting the diagnosis? |
| How did you as a family member experience finding out about the diagnosis? |
| After all this had happened, what is your everyday life like? |
| It sounds like you and/or your family member have a hard time with x….. Can you tell me about how it affects you? |
| So…. It affects you in different ways. How do you deal with it? |
| But how do you handle it when you X (X=another situation) |
| Can you tell me, is there other situations where you and your family member feel that X bothers you more than usual/is more difficult to handle? |
| What are the consequences for you? |
| In addition to these, are there other things in your life situation that you find problematic? |
| Can you tell me a little bit about how it affects you? |
| Can you tell me a concrete situation or give me a concrete example when this was a problem? |
| How did you handle it? |
| What role do other people around you play in helping you deal with the problems you are describing? |
| Would you like to tell me, what is important for you to have a decent life situation right now? |
